# The Involvement of the Small GTPase Rac1 in Insulin Signaling That Regulates Plasma Membrane Translocation of the Fatty Acid Transporter CD36 in Mouse White Adipocytes

**DOI:** 10.3390/ijms27125568

**Published:** 2026-06-20

**Authors:** Nobuyuki Takenaka, Mizuki Sakata, Yuki Abe, Kokoa Iha, Takaya Satoh

**Affiliations:** Laboratory of Cell Biology, Department of Biological Chemistry, Graduate School of Science, Osaka Metropolitan University, Sakai 599-8531, Osaka, Japan; nobu.takenaka@omu.ac.jp (N.T.);

**Keywords:** Akt2, cluster of differentiation 36, GTPase, insulin, long-chain fatty acids, Rac1, RalA, white adipocyte

## Abstract

A fraction of the insulin-stimulated uptake of long-chain fatty acids (FAs) is mediated by the FA translocase cluster of differentiation 36 (CD36) in white adipocytes. Intracellular vesicle-localized CD36 is redistributed to the plasma membrane following insulin stimulation, enhancing the uptake of long-chain FAs across the plasma membrane. We previously developed an epitope-tagged CD36 reporter, which enabled the visualization and quantification of the plasma membrane translocation of CD36. Herein, we demonstrate that the insulin-stimulated CD36 translocation is regulated by the phosphoinositide 3-kinase (PI3K)/Akt2/Rac1/RalA axis in adipocytes of subcutaneous white adipose tissue (WAT) in living mice. The uptake of long-chain FAs by insulin was completely abrogated in white adipocytes isolated from adipocyte-specific *rac1*-knockout (adipo-*rac1*-KO) mice. Correspondingly, the translocation of CD36 to the plasma membrane by insulin was also totally inhibited in Rac1-deficient white adipocytes. PI3K and Akt2 acted upstream of Rac1, and the guanin nucleotide exchange factor FLJ00068 served as a regulator for Rac1. Furthermore, the involvement of another small GTPase RalA was suggested by inhibitory effects of a dominant-negative mutant. Taken together, these results support the notion that insulin regulates the plasma membrane translocation of CD36 by mechanisms similar to those for the translocation of the glucose transporter GLUT4 in white adipocytes.

## 1. Introduction

White adipocytes take up free long-chain fatty acids (FAs) from the circulation, and utilize these FAs for the biosynthesis of triacylglycerol, which is then stored in the large intracellular lipid droplet [1,2,3,4,5]. The insulin-stimulated uptake of free long-chain FAs across the plasma membrane in white adipocytes is mediated by several types of transmembrane proteins called FA transporters, including cluster of differentiation 36 (CD36) and FA transport protein 1. CD36 also serves as a receptor for various extracellular ligands, such as oxidized phospholipids and lipoproteins, and thus belongs to the family of scavenger receptors. CD36 acts as a predominant FA transporter not only in white adipocytes, but also in cardiac and skeletal myocytes.

CD36 is composed of two transmembrane segments with a large central extracellular domain in between and short N- and C-terminal cytoplasmic tails [6,7,8]. The extracellular domain is highly glycosylated, containing a hydrophobic cavity, with which long-chain FA molecules are associated. Subsequently, these FAs are delivered into the phospholipid bilayer through CD36, and then to the cytoplasmic FA-binding protein, which is anchored to the inner surface of the membrane.

CD36 is localized not only in the plasma membrane, but also in intracellular vesicles, and the distribution of CD36 molecules between these cellular compartments is modulated by extracellular stimuli. For instance, the stimulation of cardiomyocytes with insulin promoted the net translocation of CD36 from intracellular vesicles to the sarcolemma within minutes, leading to the increase in FA uptake [7,8]. Furthermore, the redistribution of CD36 between intracellular vesicles and the plasma membrane occurred in response to insulin in in vitro differentiated adipocytes [9,10,11,12]. Hence, it is possible that insulin stimulates FA uptake through the translocation of CD36 to the plasma membrane in white adipocytes. In addition, the regulatory mechanisms underlying insulin-stimulated FA uptake by CD36 may be similar to those for glucose uptake mediated by the glucose transporter GLUT4 [12,13,14].

Recently, a novel exofacial epitope-tagged CD36 reporter (termed CD36-V5×7-GFP) was developed for monitoring cell surface translocation of CD36 in response to various extracellular stimuli [12]. Seven V5 epitope tag sequences were inserted into the extracellular domain of CD36, followed by the fusion of the green fluorescence protein (GFP) to its C terminus. This CD36 reporter is to be recognized by a specific antibody against the V5 epitope tag sequence, only when the reporter is localized in the plasma membrane, by immunofluorescent microscopy without cell permeabilization. Thus, we are able to assess the translocation of CD36 to the plasma membrane following the ectopic expression of the reporter in cells to be analyzed. By using this reporter, we previously demonstrated a pivotal role of the Rho family small GTPase Rac1 in the insulin-stimulated translocation of CD36 in 3T3-L1 adipocytes as in the case of insulin-stimulated GLUT4 translocation [12]. The involvement of phosphoinositide 3-kinase (PI3K), the protein kinase Akt2, and the guanine nucleotide exchange factor (GEF) FLJ00068 in the regulation of Rac1 downstream of the insulin receptor have also been demonstrated. Furthermore, the role of the Ras family small GTPase RalA downstream of Rac1 was described [12].

Atrophy of subcutaneous and epididymal white adipose tissues (WATs) was observed in adipocyte-specific *rac1*-knockout (adipo-*rac1*-KO) mice [13]. It has been suggested that this phenotype was caused by multiple mechanisms. First, GLUT4-mediated uptake of glucose, which was utilized for de novo synthesis of triacylglycerol, was significantly reduced in Rac1-deficient white adipocytes [13]. Second, the expression of enzymes responsible for the biosynthesis of FAs and triacylglycerol, such as ATP citrate lyase, acetyl-CoA carboxylase, FA synthase, stearoyl-CoA desaturase 1, and glycerol-3-phosphate acyltransferase 1, was remarkably suppressed through transcriptional regulation [15].

Considering that long-chain FAs transported from the circulation are also substrates for the synthesis of triacylglycerol that is stored in white adipocytes, attenuated FA uptake will likely lead to the reduction of triacylglycerol contents in the lipid droplet. Therefore, it is possible that the uptake of FAs in white adipocytes is decreased in adipo-*rac1*-KO mice, which may be another explanation for the size reduction of white adipocytes. To test this possibility, we herein investigate the effect of Rac1 KO on insulin-stimulated FA uptake and the translocation of CD36 to the plasma membrane in white adipocytes in mouse subcutaneous WAT.

## 2. Results

### 2.1. Role of Rac1 in Insulin-Stimulated FA Uptake

**Figure 1:**
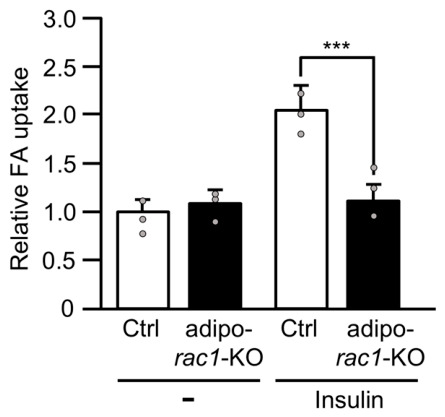
Insulin-stimulated FA uptake in primary cultured white adipocytes from control and adipo-*rac1*-KO mice. Subcutaneous WATs were isolated from control and adipo-*rac1*-KO mice, and primary cultured white adipocytes were prepared. The uptake of FA in response to insulin stimulation was measured, and the values relative to that in unstimulated cells from control mice were shown as means ± S.E. (*n* = 3). *** *p* < 0.001 (Student’s *t* test).

Previously, we demonstrated that the treatment with a Rac1-selective inhibitor or small interfering RNA (siRNA)-mediated knockdown of Rac1 completely abrogated insulin-stimulated FA uptake, suggesting that insulin signaling that regulates FA uptake in mouse white adipocytes involved Rac1 [12]. Herein, we compared insulin-stimulated FA uptake between white adipocytes isolated from control and adipo-*rac1*-KO mice (Figure 1). The uptake of FA increased approximately two-fold upon insulin stimulation in control white adipocytes. This increase was not observed in white adipocytes obtained from adipo-*rac1*-KO mice. These results further support the notion that Rac1 serves as a critical regulator of FA uptake in response to insulin stimulation in mouse white adipocytes.

### 2.2. The Signaling Pathway Composed of PI3K, Akt2, FLJ00068 and Rac1 Regulates the Translocation of CD36 to the Plasma Membrane in Response to Insulin Stimulation

The exofacial epitope-tagged CD36 reporter CD36-V5×7-GFP was previously reported [12]. We inserted seven V5 epitope tag sequences into the extracellular domain, and fused GFP to the C terminus, generating CD36-V5×7-GFP. The CD36 reporter molecules translocated from intracellular vesicles to the plasma membrane, but not those retained in intracellular vesicles, are detectable by immunofluorescent microscopy without cell permeabilization using an antibody against the V5 tag. On the other hand, the expression level of the reporter can be monitored by the fluorescent signal of GFP. Therefore, we can estimate the translocation of CD36 to the plasma membrane in response to various stimuli by calculating the ratio of V5 and GFP fluorescent intensities (V5/GFP in arbitrary units).

**Figure 2:**
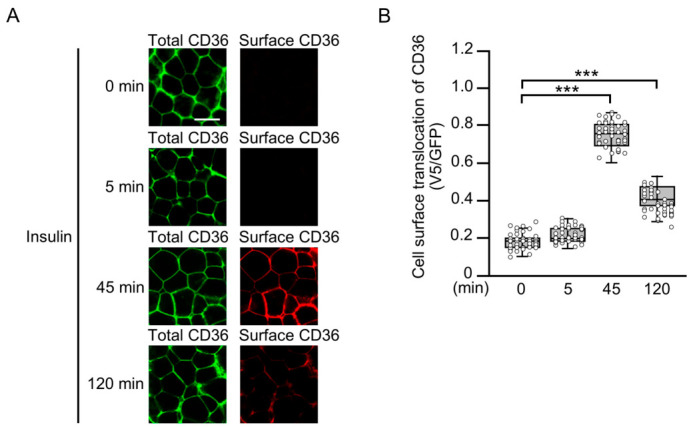
The time course of CD36 translocation to the plasma membrane induced by insulin in subcutaneous WAT from control mice. (**A**) The total amount of CD36-V5×7-GFP was estimated by the fluorescent intensity of GFP (green color). Plasma membrane-localized CD36-V5×7-GFP was visualized by immunofluorescent microscopy using an anti-V5 antibody (red color). Scale bar, 50 μm. (**B**) The translocation of CD36-V5×7-GFP to the plasma membrane shown in (**A**) was quantified. Data were analyzed using box plots (*n* = 50). *** *p* < 0.001 (Kruskal–Wallis test followed by Steel–Dwass test).

The expression plasmid encoding this reporter was introduced into subcutaneous WAT of living control mice by electroporation, and the time course of CD36 translocation to the plasma membrane following intravenous administration of insulin was examined (Figure 2). After 45 min stimulation with insulin, a significant increase in plasma membrane-localized CD36 was observed, followed by a gradual decrease over 120 min. Therefore, we routinely isolated white adipocytes at 45 min after intravenous injection of insulin to test the effect of insulin in vivo.

**Figure 3:**
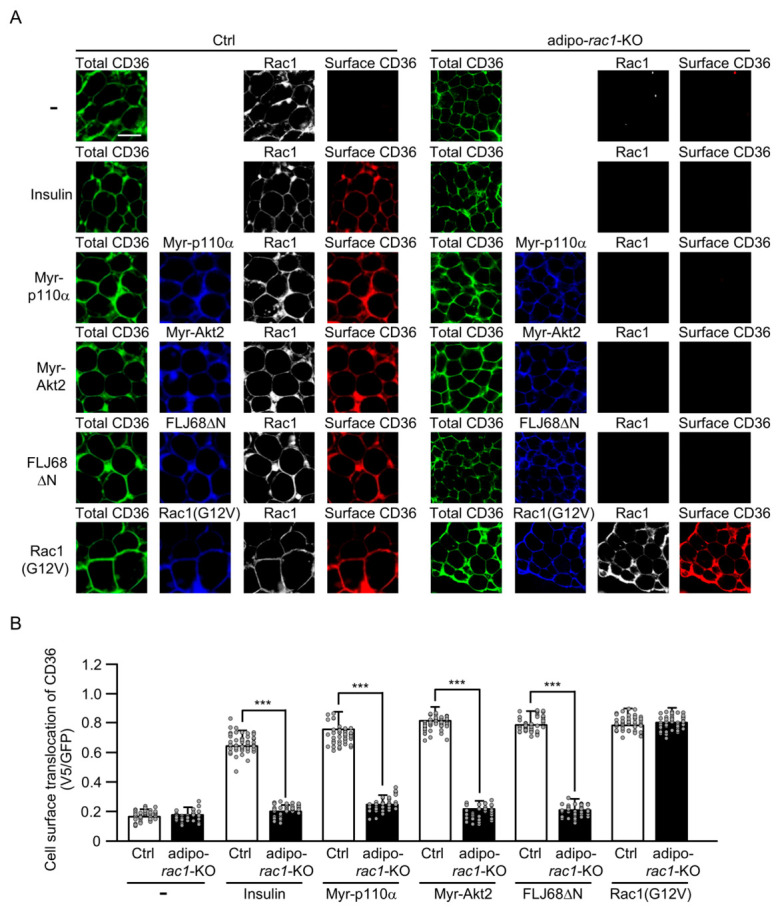
CD36 translocation to the plasma membrane induced by insulin or a constitutively activated mutant of PI3K, Akt2, FLJ00068, or Rac1 in subcutaneous WATs from control and adipo-*rac1*-KO mice. (**A**) The total amount of CD36-V5×7-GFP was estimated by the fluorescent intensity of GFP (green color). Ectopically expressed Myr-p110α, Myr-Akt2, FLJ68ΔN, and Rac1(G12V) were visualized by immunofluorescent microscopy using an anti-hemagglutinin (HA) antibody (blue color). Endogenous Rac1 and ectopically expressed Rac1(G12V) were visualized by immunofluorescent microscopy using an anti-Rac1 antibody (white color). Plasma membrane-localized CD36-V5×7-GFP was visualized by immunofluorescent microscopy using an anti-V5 antibody (red color). Scale bar, 50 μm. (**B**) The translocation of CD36-V5×7-GFP to the plasma membrane shown in (**A**) was quantified. Data are shown as means ± S.E. (*n* = 50). *** *p* < 0.001 (Student’s *t* test).

The role of Rac1 in insulin-stimulated CD36 translocation was then examined using adipo-*rac1*-KO mice (Figure 3). Insulin induced the translocation of CD36 in control, but not adipo-*rac1*-KO, mice, suggesting that Rac1 is likely to be involved in the regulation of the trafficking of CD36 downstream of the insulin receptor. On the other hand, a constitutively activated form of Rac1, Rac1(G12V), when ectopically expressed, induced CD36 translocation in both control and adipo-*rac1*-KO mice, further supporting a crucial role of Rac1. When a constitutively activated form of PI3K, N-terminally myristoylated p110α (Myr-p110α), or a constitutively activated form of Akt2, N-terminally myristoylated Akt2 (Myr-Akt2), was ectopically expressed, CD36 was translocated to the plasma membrane in control, but not adipo-*rac1*-KO, mice as in the case of insulin administration. Therefore, it is plausible that PI3K and Akt2 act upstream of Rac1, leading to the plasma membrane translocation of CD36.

The PI3K/Akt2/Rac1 axis has also been implicated in the regulation of the translocation of GLUT4 in response to insulin in white adipocytes, and the GEF FLJ00068 has been identified as a link between Akt2 and Rac1 [13,14]. Therefore, we next examined whether FLJ00068 acts as a GEF for Rac1 also in the regulation of the translocation of CD36. The expression of a constitutively activated form of FLJ00068, N-terminally truncated FLJ00068 (FLJ68ΔN), caused the translocation of CD36 in control, but not adipo-*rac1*-KO mice, supporting the notion that FLJ00068 acts as a GEF for Rac1 (Figure 3).

**Figure 4:**
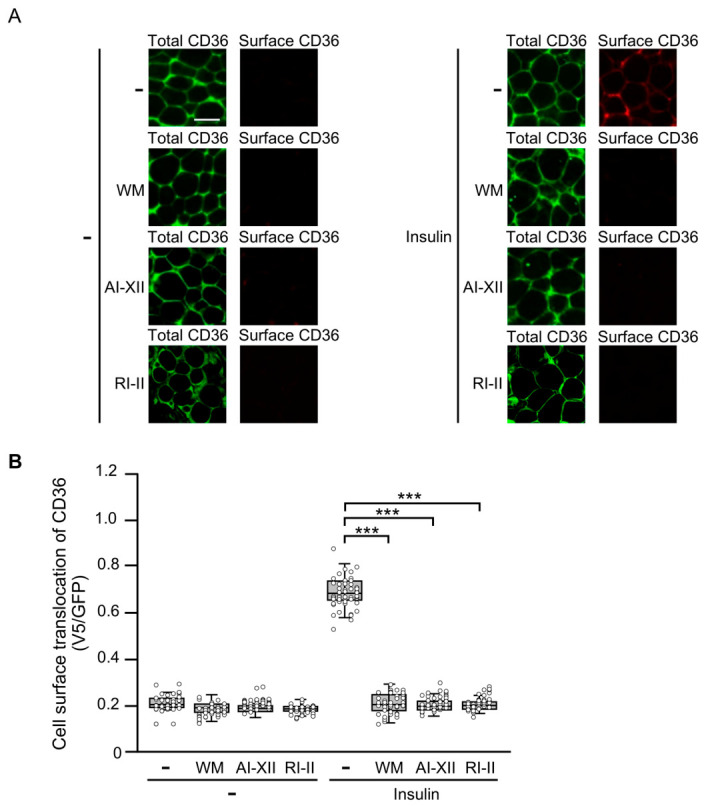
The inhibition of CD36 translocation to the plasma membrane induced by insulin in subcutaneous WAT from control mice by the PI3K-selective inhibitor WM, the Akt2-selective inhibitor AI-XII, or the Rac1-selective inhibitor RI-II. (**A**) The total amount of CD36-V5×7-GFP was estimated by the fluorescent intensity of GFP (green color). Plasma membrane-localized CD36-V5×7-GFP was visualized by immunofluorescent microscopy using an anti-V5 antibody (red color). Scale bar, 50 μm. (**B**) The translocation of CD36-V5×7-GFP to the plasma membrane shown in (**A**) was quantified. Data were analyzed using box plots (*n* = 50). *** *p* < 0.001 (Kruskal–Wallis test followed by Steel–Dwass test).

To further confirm the involvement of PI3K, Akt2, and Rac1, we then examined the effects of chemical inhibitors of these signaling proteins (Figure 4). In these experiments, subcutaneous WAT isolated from control mice was subjected to treatment with individual inhibitors prior to insulin stimulation ex vivo. Insulin-stimulated CD36 translocation was completely suppressed on the treatment with either wortmannin (WM, a selective inhibitor of PI3K), AI-XII (a selective inhibitor of Akt2), or RI-II (a selective inhibitor of Rac1). These results suggest that PI3K, Akt2, and Rac1 indeed function in the signaling pathway. Considering our previous report that insulin induced Rac1 activation in a PI3K/Akt2-dependent manner in mouse white adipocytes [13,14], it is likely that PI3K and Akt2 function upstream of Rac1 also in signaling that controls CD36 translocation.

### 2.3. Role of the GEF FLJ00068 in the Regulation of CD36 Translocation

**Figure 5:**
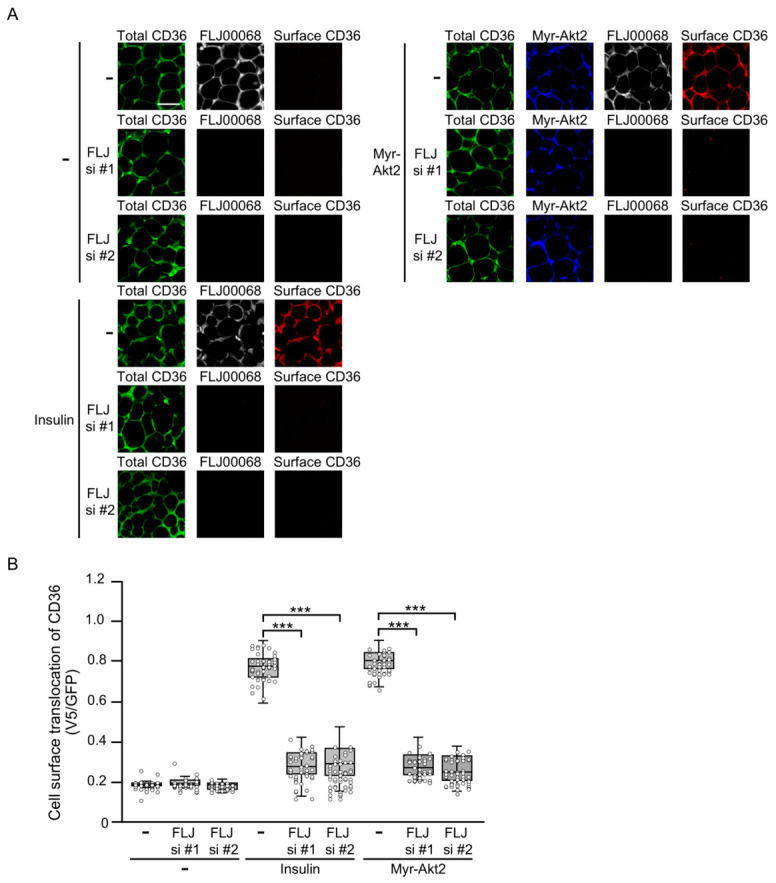
The inhibition of CD36 translocation to the plasma membrane induced by insulin or a constitutively activated mutant of Akt2 in subcutaneous WAT from control mice by siRNA-mediated knockdown of FLJ00068. (**A**) The total amount of CD36-V5×7-GFP was estimated by the fluorescent intensity of GFP (green color). Ectopically expressed Myr-Akt2 was visualized by immunofluorescent microscopy using an anti-HA antibody (blue color). Endogenous FLJ00068 was visualized by immunofluorescent microscopy using an anti-FLJ00068 antibody (white color). Plasma membrane-localized CD36-V5×7-GFP was visualized by immunofluorescent microscopy using an anti-V5 antibody (red color). Scale bar, 50 μm. (**B**) The translocation of CD36-V5×7-GFP to the plasma membrane shown in (**A**) was quantified. Data were analyzed using box plots (*n* = 50). *** *p* < 0.001 (Kruskal–Wallis test followed by Steel–Dwass test).

To further confirm the role of FLJ00068, we performed siRNA-mediated knockdown experiments (Figure 5). Two siRNA duplexes (#1 and #2) designed to reduce the expression level of endogenous FLJ00068 were employed to demonstrate its requirement for CD36 translocation. Both siRNA duplexes indeed suppressed the expression of endogenous FLJ00068 as assessed by immunofluorescence analysis. The translocation of CD36 to the plasma membrane following insulin injection or the ectopic expression of constitutively activated Akt2 was almost completely inhibited by siRNA-mediated knockdown of FLJ00068, suggesting that FLJ00068 is responsible for the regulation of Rac1 in insulin signaling.

### 2.4. Role of the Small GTPase RalA in the Regulation of CD36 Translocation

**Figure 6:**
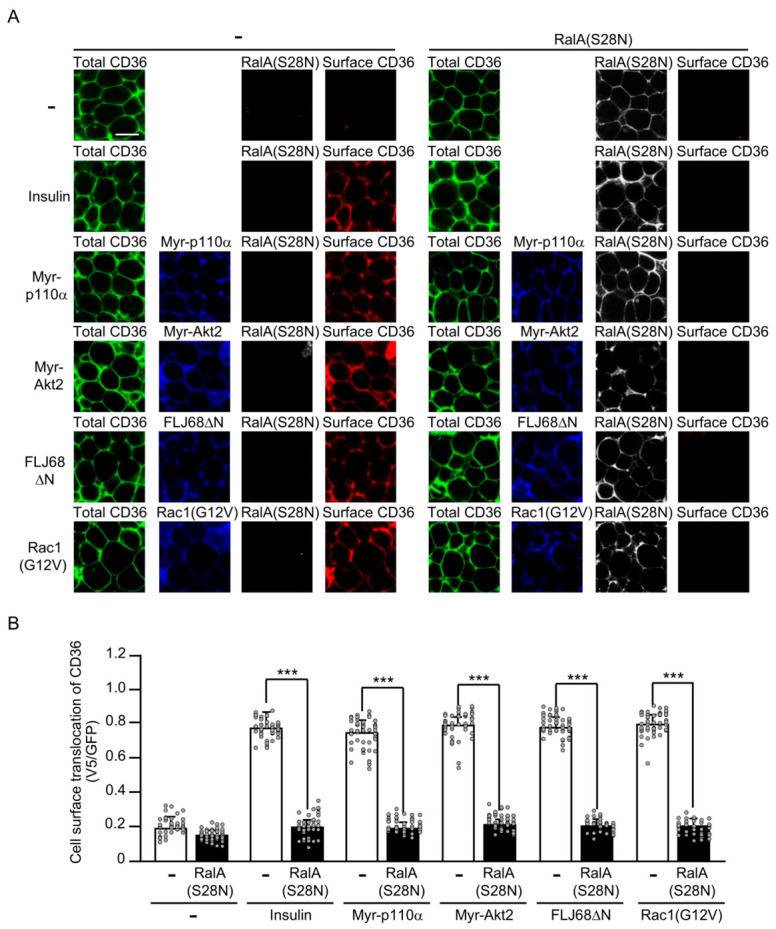
The inhibition of CD36 translocation to the plasma membrane induced by insulin or a constitutively activated mutant of PI3K, Akt2, FLJ00068, or Rac1 in subcutaneous WAT from control mice by a dominant-negative mutant of RalA. (**A**) The total amount of CD36-V5×7-GFP was estimated by the fluorescent intensity of GFP (green color). Ectopically expressed Myr-p110α, Myr-Akt2, FLJ68ΔN, and Rac1(G12V) were visualized by immunofluorescent microscopy using an anti-HA antibody (blue color). Ectopically expressed RalA(S28N) was visualized by immunofluorescent microscopy using an anti-Myc antibody (white color). Plasma membrane-localized CD36-V5×7-GFP was visualized by immunofluorescent microscopy using an anti-V5 antibody (red color). Scale bar, 50 μm. (**B**) The translocation of CD36-V5×7-GFP to the plasma membrane shown in (**A**) was quantified. Data are shown as means ± S.E. (*n* = 50). *** *p* < 0.001 (Student’s *t* test).

The Ras family small GTPase RalA has been implicated in the regulation of insulin-stimulated GLUT4 translocation both in skeletal muscle and white adipocytes [13,14,16,17]. Furthermore, the involvement of RalA downstream of Rac1 in the induction of CD36 translocation has been shown in 3T3-L1 adipocytes [12]. To test the possibility that RalA acts downstream of Rac1 also in mouse white adipocytes, we employed a dominant-negative mutant of RalA, RalA(S28N), which inhibits the activation of endogenous RalA [18] (Figure 6). The overexpression of RalA(S28N) significantly inhibited CD36 translocation in response to insulin in mouse white adipocytes. Moreover, CD36 translocation following the expression of a constitutively activated mutant of PI3K, Akt2, FLJ00068, or Rac1 was also highly sensitive to the negative effect of co-expressed RalA(S28N).

**Figure 7:**
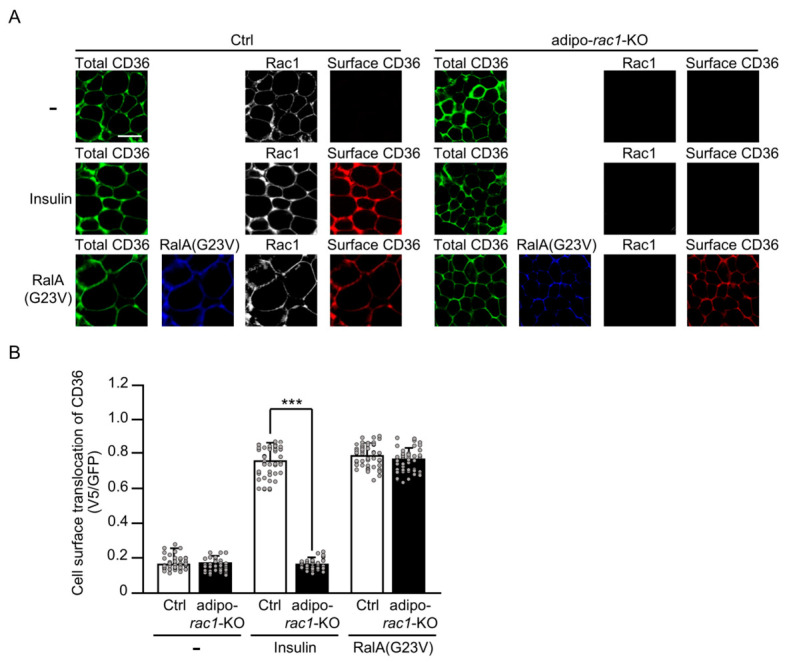
CD36 translocation to the plasma membrane induced by insulin or a constitutively activated mutant of RalA in subcutaneous WATs from control and adipo-*rac1*-KO mice. (**A**) The total amount of CD36-V5×7-GFP was estimated by the fluorescent intensity of GFP (green color). Ectopically expressed RalA(G23V) was visualized by immunofluorescent microscopy using an anti-HA antibody (blue color). Endogenous Rac1 was visualized by immunofluorescent microscopy using an anti-Rac1 antibody (white color). Plasma membrane-localized CD36-V5×7-GFP was visualized by immunofluorescent microscopy using an anti-V5 antibody (red color). Scale bar, 50 μm. (**B**) The translocation of CD36-V5×7-GFP to the plasma membrane shown in (**A**) was quantified. Data are shown as means ± S.E. (*n* = 50). *** *p* < 0.001 (Student’s *t* test).

To further confirm that RalA acts downstream of Rac1, we then examined the effect of Rac1 knockout on the plasma membrane translocation of CD36 in response to the ectopic expression of a constitutively activated mutant of RalA, RalA(G23V) (Figure 7). As expected, RalA(G23V) induced CD36 translocation in both white adipocytes of control mice and those of adipo-*rac1*-KO mice, whereas the insulin-stimulated translocation of CD36 was not observed in white adipocytes of adipo-*rac1*-KO mice as demonstrated in Figure 3.

Taken together, these results indicate that RalA regulates insulin-stimulated CD36 translocation to the plasma membrane downstream of Rac1 also in white adipocytes in living mice.

## 3. Discussion

By employing CD36-V5×7-GFP, the epitope-tagged CD36 reporter, we previously explored the mechanisms for intracellular signaling that regulates the insulin-stimulated translocation of CD36, leading to the enhanced uptake of long-chain FAs in in vitro differentiated 3T3-L1 adipocytes [12]. We demonstrated for the first time that the small GTPase Rac1 plays a pivotal role in this signaling, and PI3K and Akt2 regulate Rac1 downstream of the insulin receptor. In addition, the Ras family small GTPase RalA played an important role downstream of Rac1. It is important to ascertain whether these mechanisms indeed underlie the insulin regulation of CD36 not only in cultured 3T3-L1 adipocytes, but also in white adipocytes in living mice. Therefore, in this study, we ectopically expressed the CD36 reporter in adipocytes of subcutaneous WAT in living mice, and assessed the plasma membrane translocation of CD36 after intravenous injection of insulin. The role of Rac1 was verified by comparing adipo-*rac1*-KO mice with control mice. Moreover, the signaling cascade consisting of PI3K, Akt2, FLJ00068, Rac1, and RalA was indeed demonstrated to regulate insulin-stimulated CD36 translocation also in mouse white adipocytes. Of course, we need to recognize the inherent limitations of analyses by ectopically expressing constitutively active mutants: the possibility of excess signal input compared to physiological stimuli and failure to reflect the time course of the signal intensity in vivo. In addition, it should be noted that overexpression and tagging of the CD36 reporter may affect intracellular trafficking, leading to its properties somewhat different from those of endogenous CD36 molecules.

The regulatory mechanisms upstream and downstream of Rac1 are very similar to those for the regulation of the insulin-stimulated translocation of GLUT4 [13,14,15,16,17]. It remains unclear whether CD36 and GLUT4 reside in the same vesicles or in distinct vesicles in unstimulated white adipocytes, and future studies will be needed to determine this.

The recombinant Akt protein was reported to phosphorylate serine71 of Rac1, leading to downregulation of GTP-binding activity of Rac1 in vitro and in the human melanoma cell line SK-MEL28 [19]. However, to our knowledge, no evidence was found for direct inactivation of Rac1 by Akt2 in white adipocytes. Rather, Akt2 stimulates the downstream Rac1 signaling following insulin stimulation in these cells [12,13,14]. Although no concrete evidence has been provided, it is possible that Rac1 is not sufficiently phosphorylated by Akt2 in white adipocytes: the efficiency of Rac1 phosphorylation and consequent inactivation may be altered depending on cell types and cellular contexts.

CD36 is expressed not only in white adipocytes, but also in various other cell types, such as macrophages, monocytes, platelets, and skeletal and cardiac muscle cells, acting as a receptor for diverse extracellular ligands [1,2,3,4,5]. In cardiac muscle cells, CD36 is a major transmembrane transporter for long-chain FAs, and is also translocated from intracellular vesicles to the plasma membrane in response to insulin [7,8,20,21]. The signaling cascade composed of PI3K, Akt2, AS160, and Rab GTPases, which regulates GLUT4 translocation, has been implicated also in CD36 translocation. In addition, contraction induces CD36 translocation by mechanisms involving AMP-activated protein kinase (AMPK), which are similar to those for GLUT4 translocation. Therefore, it is plausible that the subcellular redistribution of CD36 and GLUT4 is regulated via similar intracellular signaling pathways. However, the detailed mechanisms seem to be different [20,21]. In support of this, in unstimulated cardiomyocytes, CD36 is present mainly in endosomes whereas a relatively large subcellular pool of GLUT4, referred to as GLUT4 storage vesicles, exists outside of the endosomes. Namely, CD36 and GLUT4 are primarily distributed in separate vesicle systems, although some of them are contained in the same compartments [21]. The role of Rac1 in the insulin-stimulated translocation of GLUT4 and CD36 in cardiomyocytes is unknown, and further research is needed to elucidate this issue.

Insulin induces the translocation of CD36 to the plasma membrane and the uptake of long-chain FAs not only in cardiac myocytes, but also in skeletal muscle cells [7]. Contraction also triggers these cellular responses through AMPK-dependent and -independent signaling cascades [7,22]. Considering that Rac1 has been implicated in insulin- and contraction-stimulated GLUT4 translocation and glucose uptake in skeletal muscle [14,23,24,25,26,27,28,29,30], it is possible that Rac1 also serves as a regulator for the plasma membrane translocation of CD36. Furthermore, the regulatory mechanisms involving PI3K and Akt2 may govern Rac1-dependent CD36 translocation in skeletal muscle as described for white adipocytes in this study. Additional studies will be required to clarify these issues.

In contrast to white adipocytes and cardiomyocytes, the subcellular localization of CD36 is not regulated by insulin in macrophages. CD36 recognizes and acts as a receptor for specific oxidized phospholipid moieties in oxidized low-density lipoproteins, ultimately leading to the formation of macrophage foam cells and atherosclerosis. The plasma membrane localization of CD36 is important for its function and the mechanisms underlying CD36 trafficking has been intensively explored. For instance, it has been reported that the ligand-induced phosphorylation of vimentin directed CD36 translocation to plasma membrane in macrophages [31].

As described above, the mechanisms that regulate the subcellular localization of CD36 may differ significantly depending on the cell type. The CD36 reporter that we described in this study can be ectopically expressed in virtually all types of cells in principle through gene transfer, and therefore it will be instrumental in elucidating the molecular basis of the subcellular localization of CD36 in a variety of cell types.

Adipo-*rac1*-KO mice exhibited atrophy of subcutaneous and epididymal WATs, in which white adipocytes were significantly reduced in size [13]. GLUT4-mediated glucose uptake in response to insulin was decreased in white adipocytes in adipo-*rac1*-KO mice, which may account for the smaller size of white adipocytes in these mice, because glucose is utilized as a substrate for lipid biosynthesis. Furthermore, the expression levels of various enzymes for the synthesis of FAs and triacylglycerol were decreased in white adipocytes of adipo-*rac1*-KO mice compared with the control [13]. The mechanisms whereby Rac1 regulates the expression of the genes for these enzymes were revealed at transcriptional and post-translational levels by employing in vitro differentiated adipocytes [15]. The decrease in the expression levels of these enzymes may be another cause of the size reduction of white adipocytes in adipo-*rac1*-KO mice.

Atrophy of WATs in adipo-*rac1*-KO mice may also be attributed to the defects in the insulin-stimulated uptake of long-chain FAs mediated by CD36, as reported in this study, given that long-chain FAs transported from the circulation are major substrates in triglyceride biosynthesis. It is noteworthy that the size variation of Rac1-deficient white adipocytes was observed among individual animals and even within the same WAT. Namely, it was not unusual to find normal-size white adipocytes in the WAT that exhibited overall atrophy in adipo-*rac1*-KO mice, even though the statistical analysis showed a significant reduction in size [13]. Moreover, the cell size remained normal in some cases even when insulin-responsive CD36 translocation was completely inhibited. Therefore, it is plausible that atrophy of WAT is not caused solely by the inhibition of insulin-stimulated CD36 translocation, but rather various other factors, as mentioned above, are involved.

The *rac1* gene is expected to be knocked out not only in WAT, but also in brown adipose tissue (BAT), in adipo-*rac1*-KO mice [32]. Brown adipocytes are highly specialized cells characterized by their thermogenic capacity [33,34]. Thermogenesis in brown adipocytes is mediated by mitochondrial uncoupling protein 1, whose gene expression is induced through the cyclic AMP-dependent protein kinase signaling predominantly by sympathetic adrenergic stimulation during cold exposure. Following insulin stimulation, the uptake of glucose and FA in BAT is enhanced, and thereby contributing to regulated energy expenditure and glucose and lipid homeostasis [35,36,37]. The role of Rac1 in these physiological processes in BAT is totally unknown, and will be revealed through the analysis of BAT in adipo-*rac1*-KO mice.

## 4. Materials and Methods

### 4.1. Materials

A rat monoclonal antibody against the HA epitope tag (11 867 423 001), a rabbit polyclonal antibody against the Myc epitope tag (16286-1-AP), and a goat polyclonal antibody against the V5 epitope tag (A190-119A) were purchased from Roche Applied Science (Penzberg, Germany), Proteintech (Wuhan, China), and Bethyl Laboratories (Montgomery, TX, USA), respectively. A mouse monoclonal antibody against Rac1 (610650) was purchased from BD Biosciences (San Jose, CA, USA). A rabbit polyclonal antibody against FLJ00068 (ab137898) was purchased from Abcam (Cambridge, UK). Antibodies against goat IgG, mouse IgG, rabbit IgG, and rat IgG conjugated with CF™ 350/543/647 were purchased from Biotium (Fremont, CA, USA). Insulin was purchased from Eli Lilly (Indianapolis, IN, USA). The PI3K-selective inhibitor WM, Akt2-selective inhibitor AI-XII, and the Rac1-selective inhibitor RI-II were purchased from MilliporeSigma/Merck Life Science (Burlington, MA, USA).

### 4.2. Animal Experiments

All animal experiments were approved by the Ethics Committee for Animal Experiments at Osaka Metropolitan University (Approval Code: #20-75, #21-82-2, #22-102, #23-75, #24-73 and #25-90) and carried out according to institutional guidelines of Osaka Metropolitan University. All mice used in this study are on the C57BL/6 genetic background. We routinely crossbred *rac1^flox/flox^* mice [38] with *rac1^flox/flox^*; *adipoq-Cre* (adipo-*rac1*-KO) mice to obtain adipo-*rac1*-KO mice for experiments. Adipoq-Cre transgenic mice [32] were used as controls throughout this study. Mice were fed a normal chow diet and adult (24 to 26 week-old) male mice were used for all in vivo and ex vivo experiments. PCR primers for genotyping were previously described [13].

### 4.3. Preparation of Primary Cultured White Adipocytes from Mice

Primary cultured white adipocytes were prepared from subcutaneous WATs of control and adipo-*rac1*-KO mice as described previously [12].

### 4.4. Measurement of the Uptake of FA in Primary Cultured Mouse White Adipocytes

The uptake of FA was measured in primary cultured white adipocytes prepared from control and adipo-*rac1*-KO mice as described previously [12]. Fluorescent intensities (in arbitrary units) obtained from 3 independent experiments using different primary cultures for each condition were used for statistical analysis (Student’s *t* test).

### 4.5. Gene Transfer into White Adipocytes by Electroporation

Plasmid DNAs were introduced into mouse subcutaneous white adipocytes by electroporation as described previously [13]. After mice were anesthetized [13], a combination of expression vectors (pCAGGS-CD36-V5×7-GFP, pCAGGS-Myr-p110α-HA×3, pCAGGS-Myr-Akt2-HA×3, pCAGGS-HA×2-FLJ68ΔN, pCAGGS-HA×3-Rac1(G12V), pCAGGS-Myc×7-RalA(S28N), and pCAGGS-HA×3-RalA(G23V)) (80 µg in total) and the siRNA (1.5 µg in total) dissolved in 50 µL of 9 mg/mL NaCl was injected into subcutaneous WAT with a 27-gauge needle. Following injection, electroporation was performed as described [13].

### 4.6. RNA Interference in Mouse White Adipocytes

Two types of siRNA duplexes against mouse FLJ00068, FLJ si #1 (5′-GCAACUAUGGCCACACCUUTT-3′) and FLJ si #2 (5′-CGAUUACAGGUCUGCAGUATT-3′), were purchased from MilliporeSigma/Merck Life Science. The control siRNA (1022076) was purchased from Qiagen (Venlo, The Netherlands). Either one of these siRNA duplexes was introduced into white adipocytes by electroporation as described above.

### 4.7. Detection of the Translocation of CD36 to the Plasma Membrane

The translocation of CD36 to the plasma membrane was detected essentially as described previously for GLUT4 [13]. Five days after electroporation, mice were fasted for 16 h, and insulin (175.5 µg/kg of body weight) was administered intravenously. Forty-five min later, mice were euthanized, and subcutaneous WAT was ablated from these mice. In some experiments, isolated subcutaneous WAT was stimulated with 100 nM insulin for 45 min ex vivo instead of intravenous injection following the treatment with chemical inhibitors (100 nM WM, 5 μM AI-XII, or 25 μM RI-II) for 2 h. Subcutaneous WAT was then fixed with 40 mg/mL paraformaldehyde in phosphate-buffered saline (PBS) for 30 min and were incubated with an anti-V5 tag antibody overnight at 4 °C for the detection of the plasma membrane-localized CD36-V5×7-GFP. After washing three times with PBS, subcutaneous WAT was fixed again with 40 mg/mL paraformaldehyde in PBS for 10 min, permeabilized with 0.5% (*v*/*v*) Triton X-100 in PBS for 15 min, and incubated in 0.1% (*v*/*v*) Triton X-100 in PBS supplemented with 2% (*v*/*v*) normal goat serum (G9023, MilliporeSigma/Merck Life Science) for 30 min. Permeabilized cells were further treated with anti-HA (for the detection of ectopically expressed Myr-p110α, Myr-Akt2, FLJ68ΔN, Rac1(G12V), or RalA(G23V)), anti-Rac1 (for the detection of endogenous Rac1 and ectopically expressed Rac1(G12V)), anti-FLJ00068 (for the detection of endogenous FLJ00068), or anti-Myc (for the detection of ectopically expressed RalA(S28N)) antibodies for 2 h. After washing three times with 0.1% (*v*/*v*) Tween 20 in PBS, anti-V5, anti-HA, anti-Rac1, anti-FLJ00068, and anti-Myc antibodies were detected with fluoresceinated secondary antibodies. Images were obtained and analyzed using a confocal laser-scanning microscope (FV1200, Evident (Olympus), Tokyo, Japan ). Fluorescent intensities of V5 and GFP in regions of interest were quantified using ImageJ software v1.52. The relative amount of CD36-V5×7-GFP translocated to the plasma membrane was estimated by the ratio of V5 and GFP fluorescent intensities (V5/GFP). Experiments were repeated by using three mice of each genotype. Values of 50 cells in total from 6 different images for each condition were used for statistical analysis (Student’s *t* test or Kruskal–Wallis test followed by Steel–Dwass test).

## Data Availability

The original contributions presented in this study are included in the article. Further inquiries can be directed to the corresponding author.

## Abbreviations

adipo-*rac1*-KO	adipocyte-specific *rac1*-knockout
AMPK	AMP-activated protein kinase
BAT	brown adipose tissue
CD36	cluster of differentiation 36
Ctrl	control
FA	fatty acid
FLJ68ΔN	N-terminally truncated FLJ00068
GEF	guanine nucleotide exchange factor
GFP	green fluorescence protein
HA	hemagglutinin
Myr-p110α	N-terminally myristoylated p110α
Myr-Akt2	N-terminally myristoylated Akt2
PBS	phosphate-buffered saline
PI3K	phosphoinositide 3-kinase
siRNA	small interfering RNA
WAT	white adipose tissue
WM	wortmannin
